# An *in-silico* glimpse into the pH dependent structural changes of T7 RNA polymerase: a protein with simplicity

**DOI:** 10.1038/s41598-017-06586-1

**Published:** 2017-07-24

**Authors:** Subhomoi Borkotoky, Chetan Kumar Meena, Gopalkrishna M. Bhalerao, Ayaluru Murali

**Affiliations:** 1 Centre for Bioinformatics, School of Life Sciences, Pondicherry University, Puducherry, 605014 India; 2 UGC-DAE Consortium for Scientific Research Kalpakkam Node, Kokilamedu, Tamilnadu 603104 India

## Abstract

The capability of performing an array of functions with its single subunit structure makes T7 RNA polymerase (T7RNAP) as one of the simplest yet attractive target for various investigations ranging from structure determinations to several biological tests. In this study, with the help of molecular dynamics (MD) calculations and molecular docking, we investigated the effect of varying pH conditions on conformational flexibility of T7RNAP. We also studied its effect on the interactions with a well established inhibitor (heparin), substrate GTP and T7 promoter of T7RNAP. The simulation studies were validated with the help of three dimensional reconstructions of the polymerase at different pH environments using transmission electron microscopy and single particle analysis. On comparing the simulated structures, it was observed that the structure of T7RNAP changes considerably and interactions with its binding partners also changes as the pH shifts from basic to acidic. Further, it was observed that the C-terminal end plays a vital role in the inefficiency of the polymerase at low pH. Thus, this *in-silico* study may provide a significant insight into the structural investigations on T7RNAP as well as in designing potent inhibitors against it in varying pH environments.

## Introduction

**Figure 1:**
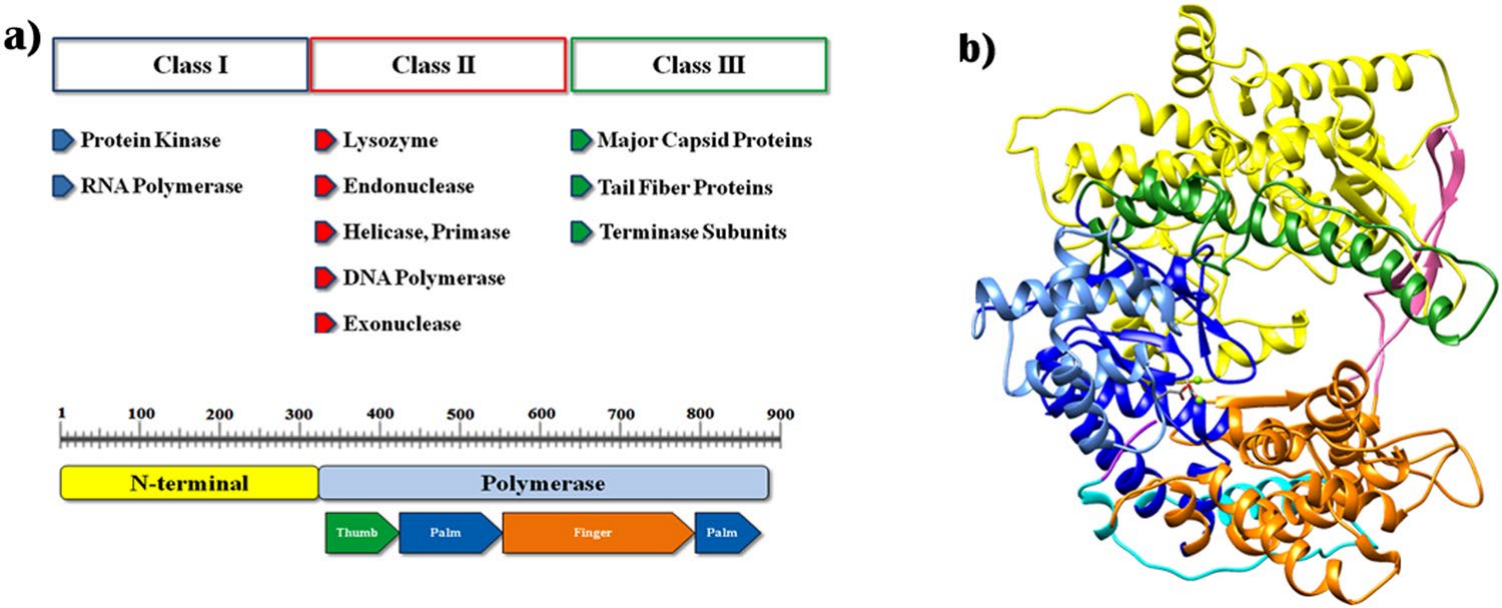
(**a**) Major gene products of T7 phage genome and domain architecture of T7RNAP with major sub-domains; (**b**) T7RNAP model structure: The color representation is as follows, the N-terminal domain (1–325), yellow; the thumb (326–411), green; the palm (412–449, 528–553, 785–879), dark blue; the palm insertion module (450–527), light blue; the fingers (554–739, 769–784), orange; specificity loop (740–769), pink; extended foot module (838–879), cyan; and C-terminal (880–883), violet. Two metal ions at the active site are shown as green spheres.

T7 bacteriophage is one of the most studied phages that has drawn the attention of many research groups due to its rapid growth and rapid adaptivity. It is an obligatory lytic phage and depends on *Escherichia coli* to replicate. The 39,937-bp genome of T7 phage is divided into 3 classes; *class I* genes establish favorable conditions for phage growth in early stage of infection, *class II* genes are involved mainly in encoding DNA replication proteins; and *class III* genes are expressed during the later stage of phage growth and mainly encode structural gene products (Fig. 1a)^1^.

The focus of this study is the 98 kDa T7 class I gene product, T7 RNA polymerase (T7RNAP). T7RNAP has an immense importance in biological research. It has been primarily used to over express heterologous genes under the control of the T7 promoter^2^, which has share in different facets of biological research ranging from industrial biotechnology^3^ to synthetic biology^4^. The interesting features of T7RNAP which attribute to its significance include its single-subunit structure in contrast to multi-subunit bacterial RNAP, high specificity towards the T7 promoter, independence of auxiliary transcription factors, efficient elongation and production of very long transcripts, termination only by class I and class II termination signals and not subject to the factors which cause termination of transcription by *E. coli* RNA polymerase.

T7RNAP consists of an N-terminal (residues 1–325) and polymerase (residues 326–883) domain. The polymerase domain of T7RNAP structure resembles the right hand similar to other nucleic acid polymerase structures^5^. The three dimensional model structure of T7RNAP with Mg^2+^ ions at the catalytic centre^6^ can be seen in Fig. 1b. The polymerase domain is further divided into sub-domains *viz*. Thumb (residues 326–411), palm (residues 412–553 and 785–879) and finger (residues 554–784) sub-domains. The C-terminal end stretch from residues ~875–883. The active site resides within the deep cleft formed by these three sub-domains. The palm sub-domain was further classified into the palm insertion module (450–527) and extended foot module (838–879) while the finger sub-domain into specificity loop (740–769) (Fig. 1b)^7^. The N-terminal domain plays a role in sequence specific promoter binding and in opening the duplex DNA. The region formed by the residues 93–101 bind the AT-rich −13 to −17 element of the promoter.

An intercalating hairpin formed by residues 232–242 inserts between the template and non template strand to open the promoter^8^. The thumb sub-domain forms a long α-helical projection on one side of the template-binding cleft. The palm sub-domain contains major catalytic residues such as Asp 537 and Asp 812^8^ as well as the residue Lys 472 which greatly promotes the PPi release^9^. The fingers sub-domain also contains many important residues: region 563–571 is involved either directly or indirectly in the interaction with the promoter; region 626–639 appear to contribute to the affinity of NTP binding; three lysine residues K711, K713 and K714 interacting with the downstream DNA are important for promoter opening^10^ and the residues 740–769 form a specificity loop that is involved promoter recognition^11^.

For exhibiting maximum activity at a certain pH, a protein needs a conformation which is optimal for its function. This conformation changes as pH deviates to either side of the optimum, leading to differences in proper function. Various studies have been conducted over time to elucidate pH dependent structural changes in various proteins with experimental as well as molecular dynamics simulations^12–17^. Since T7RNAP has a broad range of applications, the environmental effects such as temperature, pH etc. on its structure has utmost importance in T7RNAP related studies. However, there is no work done to understand the influence of these factors on T7RNAP. A recent patent^18^ on the T7RNAP mutations which can induce enhanced thermostability shows the need of residue level information for various mutational studies. The aim of this work is focused on how the T7RNAP responds to low pH and how the pH dependent conformational changes in T7RNAP influence the binding of different inhibitors and substrates. It is known that the optimal pH range for T7RNAP is 8–9^5^, and most of the experiments using T7RNAP preferred pH 7.9 or 8. A pH based activity profile of wild type T7RNAP^16^ showed a substantial enzymatic activity in the range 7.9 to 9.5, whereas diminished or no activity was observed at pH 5 in acidic range and beyond pH 11 in basic range. Since the primary focus of the present work is on the conformational changes which make the enzyme inactive at low pH, we selected pH 5. The pH 7.9 was selected as it is the optimum pH for the polymerase (also evident by the high activity in the pH activity profile^16^). Neutral pH was selected for the study as a standard between acidic and basic pH. Comparison of the circular dichroism spectra of T7RNAP at two pH values pH 5 and pH 7.9 shows that T7RNAP does not change its conformation drastically between these two pH strengths^17^. However no research is carried out to know the overall structural changes that make the T7RNAP less effective in a lower pH range.

Since molecular dynamics (MD) simulation approach (also considered as computational microscope)^19^ has the ability to capture the behavior of biological molecules in full atomic detail, we have analyzed the pH induced conformational changes in T7RNAP with MD simulation approach combined with single particle analysis^20^ as experimental validation. For more in depth analysis, few auxiliary *in silico* tools were also used to elucidate possible residue level information. Further, we have also analyzed how these conformational changes influence the interactions of T7RNAP with its inhibitor, heparin along with a substrate GTP and T7 promoter with molecular docking approach.

## Results

### Analysis of Molecular Dynamics Simulations

**Figure 2:**
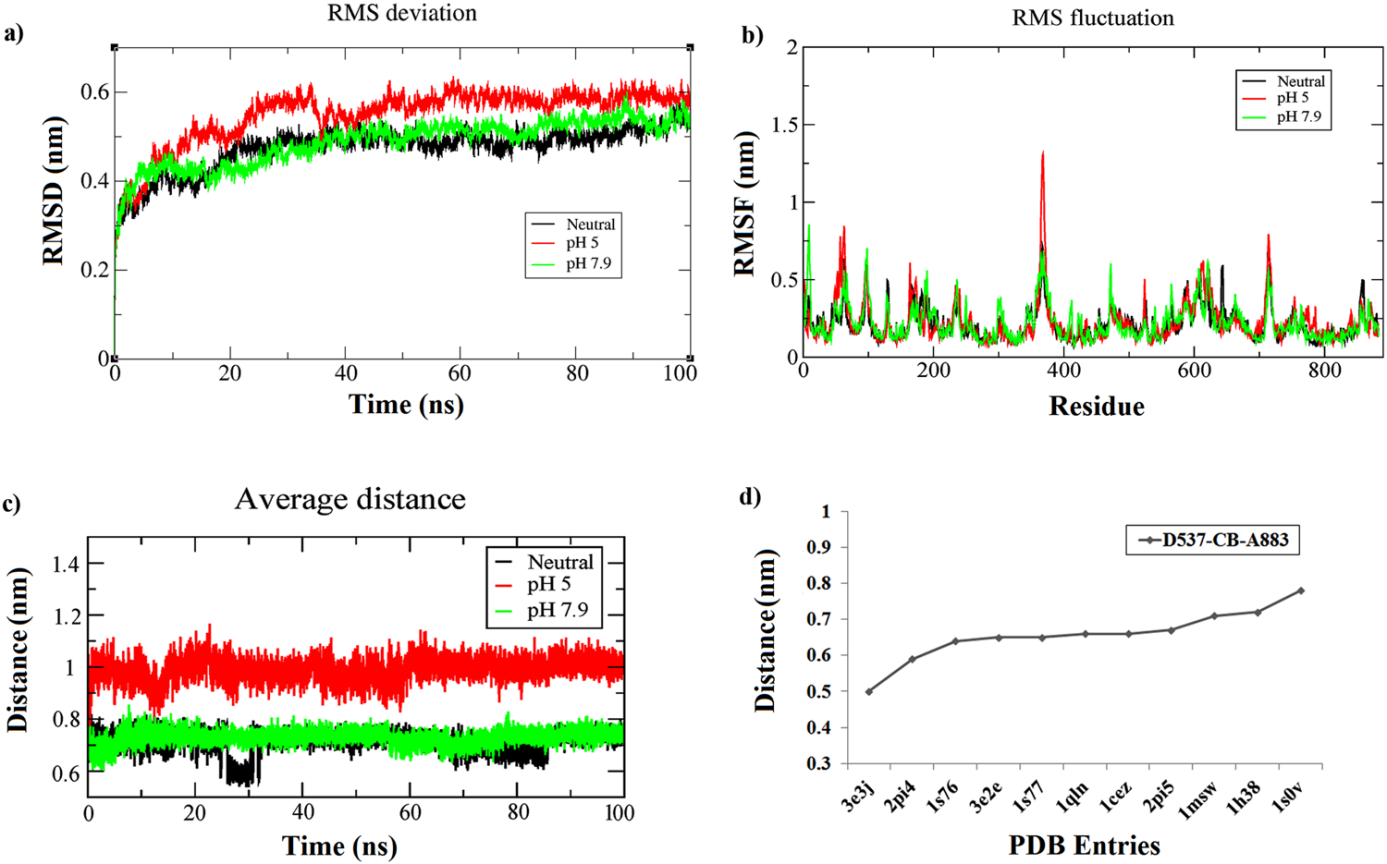
(**a**) Backbone RMSD plots for T7RNAP at three different pH values, (**b**) RMSF plots for T7RNAP at three different pH conditions. (**c**) Comparison of the average distance of C-terminal end to the catalytic core at different pH strengths, (**d**) distance of the D537 residues from C-terminal end in various experimental structures.

In order to study the pH effect on T7RNAP, we performed MD (Molecular Dynamics) simulations in different pH conditions using GROMACS (Groningen Machine for Chemical Simulations)^21^. To measure the conformational stability of the proteins after 100 ns of simulations at pH values 5, neutral and 7.9, the RMSD (Root Mean Square Deviation) profile for backbone residues were generated (Fig. 2a). The backbone RMSD varied between 0.3 and 0.6 nm during the simulation. The RMSD profile showed that, at all three pH values, the polymerase expressed stable deviation after 40 ns. It was found that during the last 60 ns of simulation period, on an average the system at pH 7.9 and neutral showed a stable deviation with RMSD about 0.5 nm whereas, at pH 5, it expressed comparatively increased deviation with RMSD about 0.6 nm.

The RMSF (Root Mean Square Fluctuation) profiles were also calculated for the protonated proteins during simulation and are shown in Fig. 2b. The profiles showed that the overall fluctuations of the protein were at maximum for pH 5 among the three pH values simulated. Further, it was noticed that a part of the thumb sub-domain region (362–375) showed the highest fluctuations for a given pH value. Other significant fluctuations were seen in parts of the N-terminal domain (57–64), and finger sub-domain (713–715).

The palm domain harbors the most catalytically critical residues (D537, D812). During simulation period, we observed overall minimum fluctuation at these residues. In case of the palm extended foot module, which takes part in lysozyme binding, highest fluctuation was observed in residues 856–858 and 860 in an order of neutral >pH 5 > pH 7.9.

The C-terminal domain (875–883) is suggested to be involved in T7 lysozyme regulation by a structural comparison between the apo-T7RNAP and T7RNAP-Lysozyme crystal structures^8^. It is believed that the C-terminal carboxylate (Ala-OH^883^), being in close proximity to T7RNAP catalytic centre, is important for metal ion-dependent catalysis by T7 RNA polymerase^7, 22^. Binding of lysozyme forces this C-terminal end to move away from its position thereby inhibits the polymerase. Figure 2c shows the average distance of the C-terminal end Ala 883 to the catalytic residue Asp 537 over 100 ns simulation period. From the figure, we can see that the C-terminal end flips away at pH 5 with an average distance of more than 1 nm from Asp 537. However, at neutral and pH 7.9 it stays closer to the catalytic centre with an average distance of ~0.7 nm, while reaching a maximum value of ~0.8 nm in case of pH 7.9. To correlate these values with the experimental results we compared 11 PDB entries of T7RNAP structures (Fig. 2d), where it was found that the distance from C-terminal A883 residue to D537 showed an average value of 0.66 nm and reached a maximum value of 0.8 nm.

### Principal component and free energy landscape analysis

**Figure 3:**
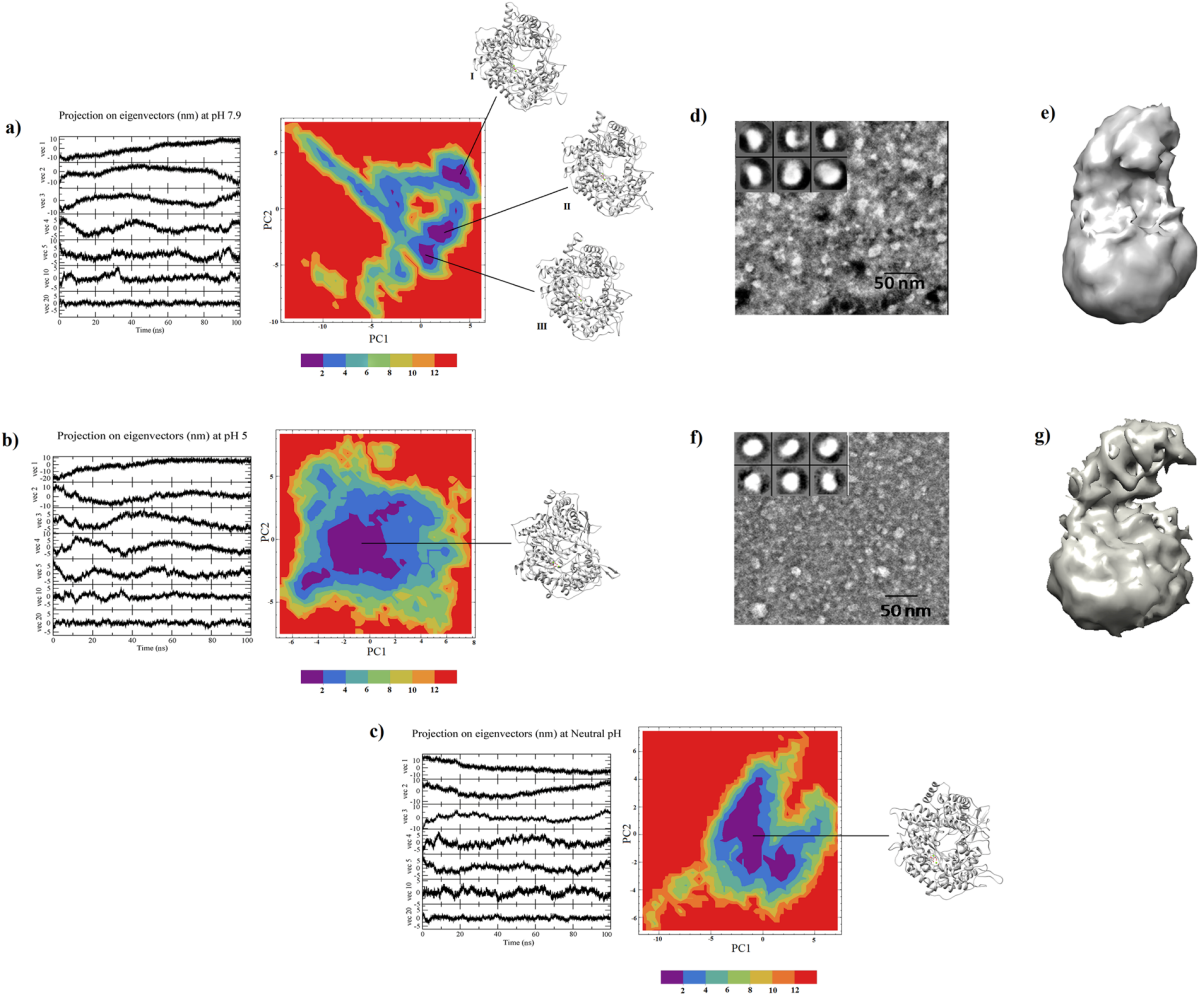
PCA analyses of T7RNAP at three different pH values depicting the motions along the first five, the tenth, and the twentieth eigenvectors during the 100 ns simulation run and FEL analyses of T7RNAP depicting low energy basin along with the representative structure retrieved at each pH. (**a**) pH 7.9, (**b**) pH 5 and (**c**) neutral pH. SPA analysis on T7RNAP at pH 7.9 and 5 showing sections of TEM micrographs of negatively stained T7RNA (**d**) at pH 7.9 and at (**f**) pH 5 (inset shows the representative views of the average of several particles – not to scale) with their respective 3D reconstruction views (**e)** and (**g)** respectively.

A Cartesian coordinate based Principal Component Analysis and Free Energy Landscape analysis was performed to analyze the conformational dynamics and to extract minimum free-energy structures of T7RNAP at different pH environments. Since first few eigenvectors capture majority of the internal motions, we retrieved the first five, the tenth, and the twentieth projections from the protein trajectories at each pH during 100 ns simulation and projected them onto the eigenvectors as obtained from the covariance matrices (Fig. 3). Steep curves of eigenvalues were obtained after plotting eigenvalues against the eigenvectors at each pH (Supplementary Fig. S1a), and it was observed that 90% of the backbone motion was covered by the first 20 eigenvectors. These results indicated that the motions of the backbone reached their equilibrium fluctuations in the first ten eigenvectors. The trajectories were projected onto the planes defined by two eigenvectors (the tenth and twentieth eigenvectors) from the backbone coordinate covariance matrix for each pH (Supplementary Fig. S1b). The projections of these two eigenvectors onto the plane of the backbone motion at each pH are strongly correlated, and fill the expected ranges almost completely which indicates that there is no high projection observed far from the diagonal. After analyzing the first 20 projection eigenvectors at each pH for their cosine content^23^, the principal components (PCs) with cosine content less than 0.2 were used as suitable reaction components, PC1 and PC2, to construct the free energy landscape (FEL) contour maps at individual pH.

The contour map at pH 7.9 (Fig. 3a) showed three different energy clusters namely I, II and III depicting the structural transition to distinct active conformational states. The low energy representative structures were retrieved at 40 ns, 68 ns and 82 ns respectively. Since the clusters II and III were found in the same plateau of the FEL with a smaller RMSD (1.22 Å) and cluster II being comparatively larger than cluster III, we selected the representative from cluster II (68 ns) and cluster I (40 ns) for further analysis.

From the contour map at pH 5 (Fig. 3b) and neutral (Fig. 3c), coordinates were collected from the largest minimum energy clusters, and lowest energy representative structures corresponding to the coordinates at 72 ns and 64 ns were retrieved respectively.

### Single particle analysis (SPA) reconstructions

From the micrographs obtained at pH 7.9, about 6,000 particles were collected using *boxer* program and filtered and centered. Class averages were generated for this particle data set without imposing any symmetry (Fig. 3d). The final model (Fig. 3e) was converged to 29 Å resolution (as measured using FSC 0.5 criterion) (Supplementary Fig. S2a).

At pH5, we obtained about 5,000 particles of T7RNAP. Class averages were generated without imposing any symmetry (Fig. 3f). The final model (Fig. 3g) was constructed at 32 Å resolution following the FSC 0.5 criterion (Supplementary Fig. S2b).

For further analysis of pH dependent conformational changes, individual structures of T7RNAP at both pH 7.9 and pH 5 were required. Hence the density maps obtained in the SPA study were used as a measure to select suitable conformations. The two representatives obtained from the FEL analysis at pH 7.9 (cluster I and cluster II) were fitted to the low resolution density map (Supplementary Fig. S2a, right panel) using rigid fitting module of Modeller^24^. The cross-correlation score for representative I was found to be 0.7443 and for representative II, it was 0.7459. Representative II (at ~ 68 ns) was favored by both EM density map fitting as well as a stable region in the RMSD profile (Fig. 2a) and hence was considered for the next step in the analysis.

The representative of FEL collected at pH 5 was fitted to the density map using rigid fitting. The cross-correlation score was found to be 0.7516. These results support the simulation studies at their respective pH strengths.

### Domain motion analysis

**Figure 4:**
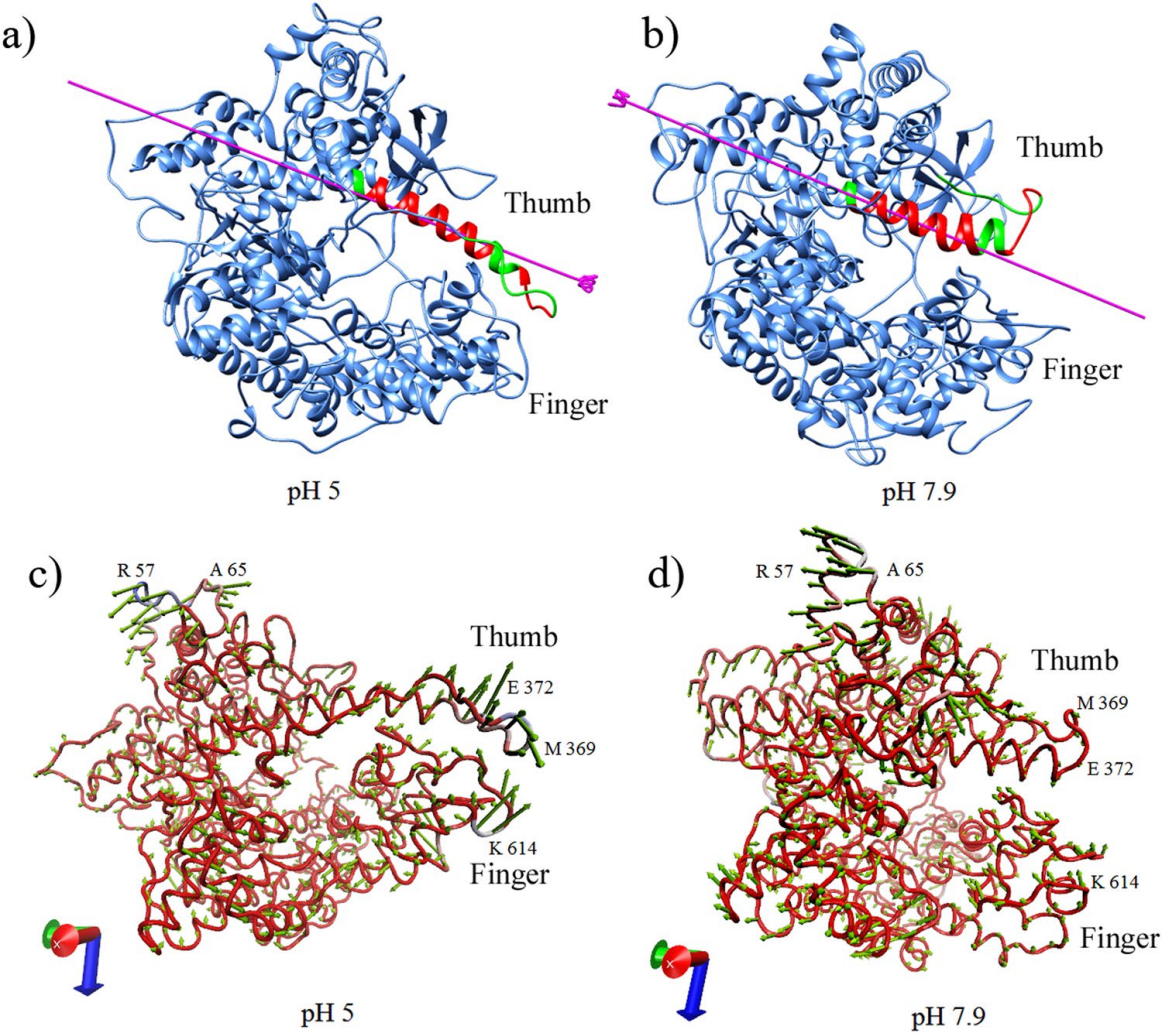
Domain motion analysis showing thumb sub-domain movement of T7RNAP as it transitions from pH 5 (**a**) to pH 7.9 (**b**) as identified by Dyndom. The hinge regions are colored red and the moving domains are colored green. The direction of movement along the hinge axis is depicted by the pink arrow. Porcupine plots showing prominent motions with directions at (**c**) pH 5 and (**d**) pH 7.9.

In order to find any domain motion among the conformations at three pH strengths, the stable conformations of T7RNAP obtained from FEL study were examined with respect to one another using DynDom^25^ analysis. No noticeable domain motions were detected according to DynDom criteria between the selected representatives at pH 5 and neutral pH or neutral pH and pH 7.9. However, domain motions between the representatives at pH 5 and pH 7.9 were observed. The residues of thumb sub-domain 361–368, 375–378 and 395–396 showed bending, whereas the residues 369–374, 379–394 acted as hinge (Fig. 4a and b). The movement of thumb sub-domain towards the finger sub-domain with a closure property of 72.1% was mediated by both translational (8.3 Å) and rotational motions (132.8°) with respect to the hinge residues. The parameter closure property (CP) can be used to term it as closure or twisting motion (CP > 50%: closure; CP < 50%: twisting)^26^.

In order to visualize the direction of movements captured by the eigen vectors, porcupine plots were generated using the extreme projections of principal component for both pH 5 and pH 7.9. The porcupine plot analysis at pH 5 (Fig. 4c) showed projections with strong outward motions in the thumb sub-domain region (comprising of amino acid residues 361–396 constituting one loop and one helix) as well as in the finger sub-domain (a region comprising of amino acid residues 612–615) bringing them close to each other. But, these motions were negligible at pH 7.9 (Fig. 4d). Other significant motions observed in both pH values 5 and 7.9 were at the N-terminal region (52–65) with strong outward motions which were also seen with strong fluctuations in the RMSF plot (Fig. 2b).

### Structural transition in T7RNAP

**Figure 5:**
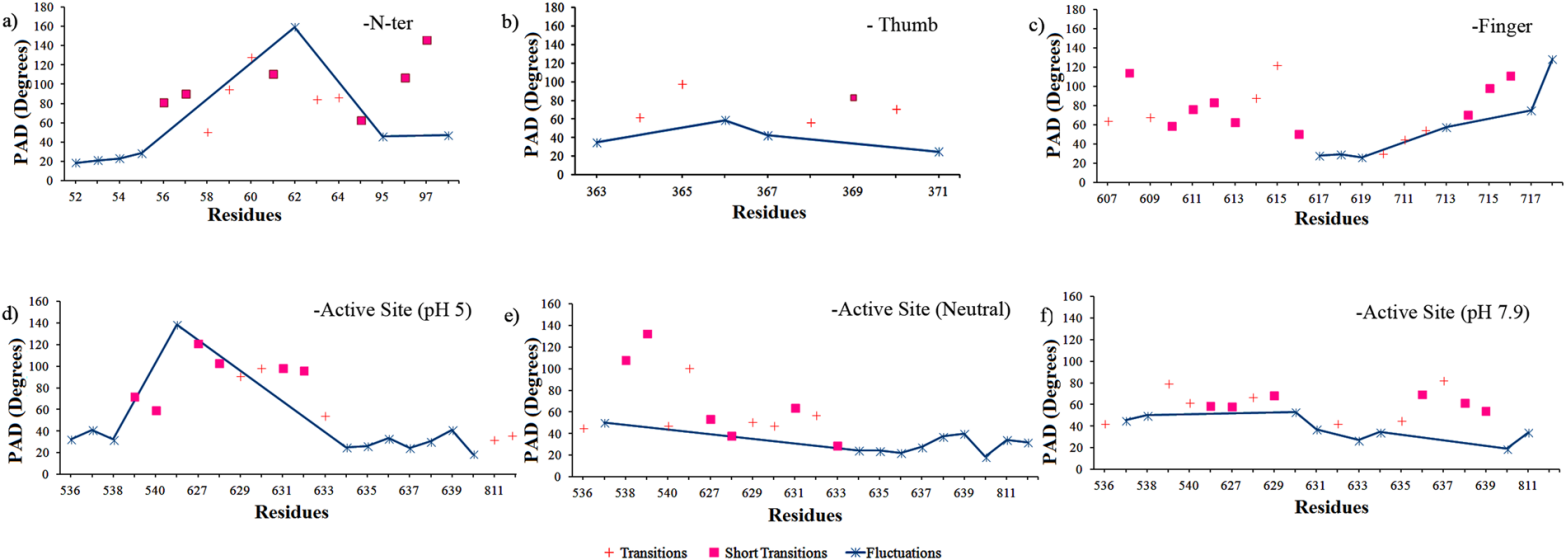
The residue fluctuations, transitions and short transitions of selected regions of T7RNAP calculated using T-pad tool and plotted based on the residue and their PAD degree from 100 ns MD simulation. The local fluctuations identified from the regions with high RMSF at pH 5 were shown for (**a**) N-terminal and (**b**) Thumb sub-domain and (**c**) finger sub-domain. The plots: (**d**,**e** and **f**) shows local fluctuations in the residues in vicinity of active site region at, pH 5, neutral pH and pH 7.9 respectively.

Understanding the local fluctuations and conformational transitions in proteins helps in identifying the flexible and rigid residues within a protein that may play a probable role in regulating various molecular recognition processes. The protein angular dispersion (T-pad^27^) tool gives a quantitative description of the intrinsic plasticity of each residue by identifying the local residual fluctuation (F), transition (or long transition, T) and short transitions (t) based on the protein angular dispersion angle ω (PADω). From the RMSF plot (Fig. 2b), it was observed that few residues of N-terminal domain, thumb sub-domain and finger sub-domain regions have higher overall atomic fluctuations. Hence, we subjected the MD trajectory at pH 5, including the highly fluctuating residues, for T-pad analysis to get an insight into the local fluctuations and conformational transitions (Fig. 5 and Supplementary Tables 1 and 2).

The residues of N-terminal domain 52–55, 95 and 98 showed fluctuations with PAD degree range 18° to 47° and residue 62 showed highest fluctuations with PAD degree 159°. The residues 56–57, 61, 65 and 96–97 showed short transitions, out of which residue 97 being highest 146.13°. The residues 58–60 and 63–64 showed long transitions, where the residue 60 has the highest PAD degree 128° (Fig. 5a).

In case of thumb sub-domain, the residues 363, 366–367 and 371 had a PAD degree range of 25° to 59° and the residues 364–365, 368 and 370 had long transitions with a PAD degree range of 56° to 98° (Fig. 5b).

The finger sub-domain residues 617–619, 713 and 717 showed fluctuations with PAD degrees ranging from 28° to 76° and residue 718 being the highest fluctuating residue with PAD degree 132°. The residues 608, 610–613,616 and 714–716 showed short transitions; where the residue 608 made a short transition with highest PAD degree 114°. The long transitions were seen at the residues 607, 609, 614, 615 and 620, where the residue 615 has a highest PAD degree 122° (Fig. 5c).

These local fluctuations were also seen in active site region and the residues in its vicinity. It was found that at pH 5 (Fig. 5d), the residues Asp 537, Tyr 639 showed fluctuations 41° and 40° respectively. Lys 631 had a short transition with PAD degree 98° while the residues His 811 and Asp 812 have shown long transitions with PAD degrees 32° and 36° respectively. In contrast, neutral pH (Fig. 5e), the active site residues Asp 537, Tyr 639, His 811, Asp 812 showed fluctuations with PAD degree 50°, 40°, 34° and 32° respectively and Lys 631 had a short transition with PAD degree 64°. At pH 7.9 (Fig. 5f), the active site residues Asp 537, Lys 631 and His 811 had fluctuations with PAD degree 45°, 37° and 34° respectively. While the other active site residues like Tyr 639 had short transition with a PAD degree 54°, the Asp 812 had a long transition with PAD degree 46°.

Among other residues near the vicinity of active site residues in the region 626–639, the residue Lys 627 which contributes to the affinity of NTP binding had shown short transitions with PAD degrees 54° and 58° at neutral pH and pH 7.9 respectively whereas strong short transition with PAD degree 121° was observed at pH 5. Another important residue Met 635, which has a role in making interactions with the ribose moiety, had shown similar fluctuations with PAD degree 24° and 26° at neutral pH and pH 5 respectively while at pH 7.9 it showed long transition with PAD degree 45°.

### Interactions with heparin and GTP

The interaction analysis of the inhibitor heparin to the individual representatives of T7RNAP at different pH values (Table 1) revealed that, heparin binds strongly to the T7RNAP structure at pH 7.9 and neutral pH with similar docking scores: −10.96 kcal/mol and −11.01 kcal/mol respectively; while the pH 5 counterpart showed comparatively much lower docking score: −7.63 kcal/mol. This indicates that the structural changes at the heparin binding sites of pH 7.9 and neutral pH are almost same, whereas there is much change at pH 5 that contributed to a lower docking score. The 2D representations of docked complexes are shown in Supplementary Fig. S3. The diagrams were generated by LigPlot^+^ tool^28^.

**Table 1:** Docking results of complexes of T7RNAP at different pH with heparin and GTP.

	Heparin		GTP	
	Docking Score (kcal/mol)	Interacting residues	Docking Score (kcal/mol)	Interacting residues
T7RNAP (pH 7.9)	−10.96	Thr 127, Ser 128, Asp 130, Asp 421, Arg 423, Gly 424, Gln 435, Gly 436, Asn 437, Lys 441, Phe 536, Asp 537, Arg 632, Ser 633,Tyr 639, Gln 649, Glu 652, Ile 810, His 811, Asp 812.	−9.51	Ser 128, Arg 394, Gly 424 Gln 435, Gly 436, Asn 437, Asp 537, His 811, Asp 812, Ser 813.
T7RNAP (pH 5)	−7.63	Arg 425, Lys 441, Asn 437, Asp 438, Asp 471, Arg 478, Asp 506, Cys 510, Asp 537, Asp 569, Ser 541,Tyr 571, Thr 630, Lys 631, Arg 632, Met 635, His 784, Gln 786, Asp 812.	−7.30	Asp 471, Asp 537, Gly 538, Ser 539, Tyr 571, Thr 630, Lys 631, Arg 632.
T7RNAP (Neutral)	−11.01	Gly 436, Asn 437, Asp 438, Asn 466, Val 470, Arg 478, Asp 506, Ser 507, Cys 510, Tyr 571, Lys 631, Arg 632, Met 635, Glu 652, Asp 653, His 811, Asp 812.	−9.11	Lys 441, Asn 466, Asp 471, Lys 472, Arg 478, Cys 510, Ala 535, Arg 627, Ile 810, His 811.

On analyzing the binding affinity of GTP to T7RNAP representatives extracted from the minimum energy clusters at different pH values (Table 1), it was observed that GTP binds strongly to T7RNAP representatives with docking scores of −9.51, −9.11 kcal/mol and −7.30 kcal/mol at pH 7.9, neutral and pH 5 respectively. The 2D representations of docked complexes are shown in Supplementary Fig. S4.

### Interactions with promoter

After docking the representative structures of T7RNAP at different pH strengths with promoter in HADDOCK server^29^, the best interaction models (Table 2) were selected based on HADDOCK score and energies (internal energy complex and binding energy) and compared interaction pattern of T7RNAP-promotor complex (PDB ID: 1CEZ)^30^. The T7 promoter interacted with T7RNAP at pH 7.9 with highest HADDOCK score (−117.1 ± 11.9) and internal energy (−33092) as compared to its counterparts. The binding energy is also found to be good (−48158.4). The pH 5 and neutral pH representatives of T7RNAP were found to be interacting with the promoter with much lower HADDOCK scores −78.4 ± 20.9 and −72.8 ± 8.3 and internal energies, −32150 and −30888 respectively. The PDBePISA interface analysis^31^ was carried out to investigate the interaction energy of the docked complexes. The analysis showed the complex of T7RNAP (pH 7.9) with promoter to have solvation free energy of −19.5 kcal/mol whereas the complexes of T7RNAP (pH 5) with promoter and T7RNAP (Neutral) with promoter were observed to have energies of −17.4 and −12.1 kcal/mol, respectively.

**Table 2:** HADDOCK docking results for the complexes of T7RNAP at different pH and T7 promoter. The HADDOCK scores are in arbitrary units (a.u.). Solvation free energy interface calculated using PDBePISA.

Complex	Haddock Score	Binding energy	Internal energy complex	Solvation free energy interface (∆^i^G, kcal/mol)
T7RNAP + Promoter (pH 7.9)	−117.1 ± 11.9	−48158.4	−33092	−19.5
T7RNAP + Promoter (pH 5)	−78.4 ± 20.9	−52160.8	−32150	−17.4
T7RNAP + Promoter (Neutral pH)	−72.8 ± 8.3	−52191.8	−30888	−12.1

**Figure 6:**
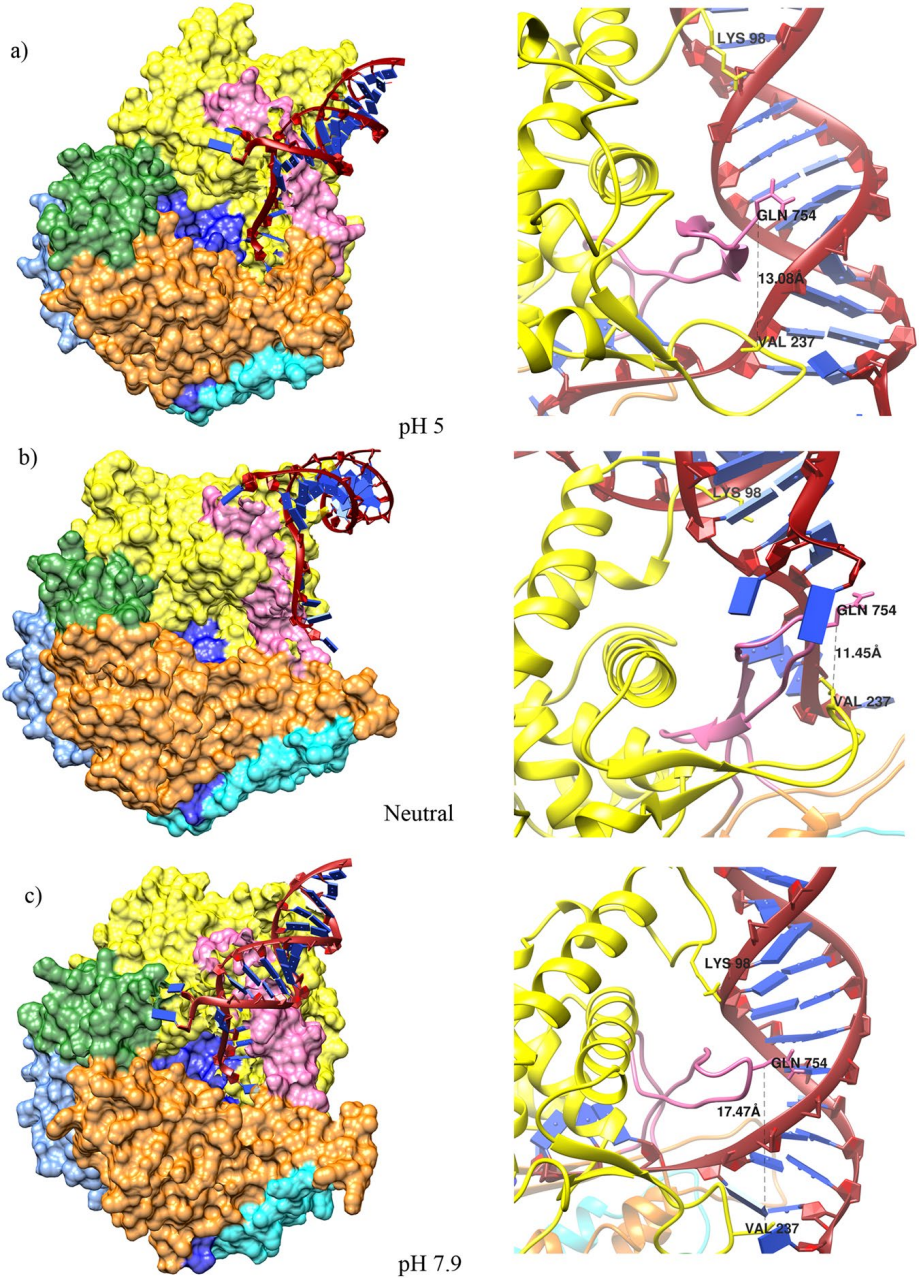
The docked complexes of T7 promoter DNA and T7RNAP at different pH strengths (**a**) pH 5, (**b**) Neutral and (**c**) pH 7.9. The color representation for T7RNAP was kept same as in Fig. 1. The right panel shows a close up view of the interacting loops in each pH.

From the docked complexes (Fig. 6), it was observed that the two loops known to interact with the T7 promoter [β-hairpin loop (232–242) and specificity loop (740–769)] have a good amount of space between them (17.47 Å). However, reduced spacing were observed at pH 5 (13.08 Å) and neutral (11.45 Å).

The MD simulation and post simulation analysis (e.g. domain motion analysis, T-PAD analysis etc.) showed that the T7RNAP structure undergoes few conformational changes. These changes were also seen to play a role in the interaction differences with the binding partners (e.g. GTP, heparin, T7 promoter) of T7RNAP.

## Discussion

For proper functioning of a protein, the pH is considered to be a major factor and the functional characteristics of a protein can be directly correlated to its structure. Hence, we employed various *in silico* tools to understand the stability and nature of conformational transitions of T7RNAP at different pH strengths.

To obtain the representative structures at different pH strengths, we used MD simulation and FEL analysis in conjunction with TEM & single particle analysis. The fitting studies of the simulated structures with the density maps at their respective pH showed a good correlation. The MD simulation results showed that T7RNAP at pH 5 displayed comparatively more dynamism than at pH 7.9 and neutral counterparts as evident from the RMSD and RMSF plots.

A flip away activity was observed in the C-terminal in pH 5 with a difference of more than 0.2 nm. As we know from the T7 lysozyme inhibited structure of T7RNAP that flipping out of the C-terminal attributes to lower activity of T7RNAP, we can propose that this distance may contribute to lower activity of T7RNAP at pH 5. This observation was also supported by the docking studies with GTP, where we can see that the complexes at pH7.9 and neutral pH show a strong binding score as compared to pH 5. The known inhibitor heparin also binds in a similar fashion to T7RNAP in the given pH values. The interacting residues found at pH 7.9 such as Gln 435, Gly 436, Asn 437, His 811 and Asp 812 were also found to be interacting in the crystal structures of T7RNAP with GTP (PDB IDs: 2PI4^32^ and 1QLN^33^), which adds to accuracy of the docking results (Table 1). The complex at neutral pH also contains few of these residues, while they were missing in the T7RNAP-GTP complex at pH 5. Although we did not see any major fluctuations in the active site residues in the RMSF plot, differences in their PAD degrees at all the pH values were observed. These differences might have contributed to the binding differences at the respective pH values.

The simulation results showed that the thumb sub-domain region (362–375) had the highest fluctuations. Out of these, the residues Pro 364, Glu 365, Met 369 and Asn 370 exhibited high transitions in backbone whereas the residue 366 had a high fluctuation propensity as evident from the PAD degrees calculated from pH 5 simulation trajectory. Other major fluctuations were seen in N-terminal domain and finger sub-domain. The residues in N-terminal such as 57, 59–61 and 96–97 showed strong transitions, while the residue Glu 62 had strong fluctuation. The residues 96–97 were involved in T7 promoter binding as observed in T7RNAP-T7L promoter crystal structure. The impact of strong transition in these residues on T7 promoter binding can also be seen in the T7RNAP-T7L promoter docking studies, where Arg 96 and Lys 98 were unable to make interactions (Fig. 6). In case of finger sub-domain, the residues 607–615 and 714–716 showed strong backbone transitions, while residues 717 and 718 showed strong fluctuations. Strong backbone transition at Lys 714, part of the positively charged patch (K711, K713 and K714), at pH 5, might impede the interaction with DNA. The residues Arg 720 to Ala 724 were seen taking part in non-bonded interactions in the T7RNAP-T7L complex (PDB ID: 1ARO) (Supplementary Fig. S5), hence strong fluctuations in the residues Glu 717 and Ile 718 may influence the interaction pattern and contribute to the T7RNAP and lysozyme binding differences.

During domain motion analysis, thumb sub-domain region (aa 361–394) was seen showing major dynamism leading to a closure motion towards finger sub-domain. This movement may also affect the efficiency of T7RNAP in lower pH values. With the T-pad analysis results, it can be deduced that the residues 364–366 and 369–370 have the major contribution to this dynamism. It was suggested that the thumb sub-domain region (aa 345–385) is a flexible element and it might form a clamp upon binding DNA^8^. Hence, it can be assumed that the movement of thumb sub-domain may affect the formation of clamp at pH 5, which in turn can influence its binding of DNA.

From the T-pad analysis of the active site residues (Fig. 5), it was observed that the catalytic and passive residues at the active site of T7RNAP at pH 5 showed highest transitions. Its effect was also directly observed in the interaction of GTP and heparin to the T7RNAP at different pH strengths (Table 1).

From the interaction pattern (Fig. 6 & Supplementary Fig. S6), it was obvious that the conformation of T7RNAP attained at pH 7.9 is more suitable for T7 promoter binding. The complex of T7RNAP with promoter at pH 7.9 made the major interactions in contrast to other two complexes. The interactions made by residues such as Arg 96 and Lys 98 located in the AT-rich recognition loop and Val 237 at the tip of this β-hairpin loop of N-terminal are important for T7RNAP-promoter complex. The interaction made by residue Asn 748 of the finger domain specificity loop (which is the only major groove interaction with bases of non-template strand) and interaction of Arg 756 with the template strand at base pair G-9 are essential for recognition of T7 promoter, as evident in the crystal structure of T7RNAP-promoter complex^30^. A lower space between the two promoter interacting loops (at pH 5) also hampers the proper interaction (Supplementary Fig. S4) and movement of the template strand. This hypothesis was validated by the comparison of six PDB entries of T7RNAP complexes where distance ranging from 15 Å to 24 Å was observed (Supplementary Fig. S7).

In conclusion, with the help of different computational approaches, it was observed that there are no major structural differences in T7RNAP at both pH5 and pH 7.9 in accordance with experimental studies^17^. However, the low processivity of T7RNAP at lower pH (in the current case pH 5) depends mainly on the minor changes in following regions: N-terminal domain (93–101 and 232–242), thumb sub-domain (362–375), finger sub-domain (713–718 and 740–769) and the proximity of C-terminal to the palm domain in addition to minor changes within the catalytic region. While most of the experimental structure determination techniques do not give the conformation of the protein in the lowest energy state that it would have in solution, these *in silico* dynamics simulation at different pH conditions and docking studies can provide additional structural and functional insights into the study of T7 RNA polymerase. However, like any other computational *in-silico* predictions, further experimental validations such as mutational studies on the obtained results may help in making T7RNAP effective in low pH environments as well as in designing potent inhibitors against it in varying pH environments.

## Methods

### Molecular dynamics simulation

Though experimentally determined structure of T7 RNA polymerase in its apo form is available in Protein Data Bank, PDB ID: 4RNP^34^, the reported structure contains only α-carbons with a considerable amount of residues without corresponding structure. As the focus of this study is pH dependence and many of these unaccounted sequences contain titrable residues, we have used the modelled full-length structure of T7RNAP, determined by homology modelling with Mg^2+^ ions at the catalytic centre^6^.

In order to study the effect of pH on T7RNAP, the model was subjected to exhaustive MD simulation up to 100 ns with GROMACS 4.5 simulation package^21^ under three different pHs *viz*. 5, neutral and 7.9. The H++ server^35^ was used to prepare the series of protonated proteins. This server automates the process of preparing the input files for typical molecular dynamics simulations. The topology for pH 5, pH 7.9 and neutral T7RNAP were generated using pdb2gmx command of GROMACS by setting the protonation and de-protonation states of K, R, D, E and H residues as identified using H++ server. For the T7RNAP representatives at pH 5 and pH 7.9, the net charge of the system was maintained after the protonation and de-protonation step, while the neutral structure was neutralized by replacing the water molecules with CL^−^ & NA^+^ counter ions based on their net charge. The force field parameters were assigned according to Gromos force field^36^. The SPC water model was used to solvate the system, which was generated as a cubic box like area with a side of 1.5 nm such that the protein is covered appropriately with water molecules. Energy minimization was performed using the steepest descent method for 50,000 steps for all systems with a tolerance of 1000 kJ mol^−1^nm^−1^. Consequently, 50,000 steps of a conjugate gradient algorithm was also used to minimize the systems with a tolerance of 1000 kJ mol^−1^ nm^−1^. For long-range interactions, the PME method was used with a 1.0 nm cut-off. Then, equilibrations were carried out for 100 ps for each system with NVT (constant number of particles, volume, and temperature) with modified Berendsen thermostat with velocity rescaling at 310 K and a 0.1 ps time step, Particle Mesh Ewald coulomb type for long-range electrostatics with Fourier spacing 0.16 followed by NPT (constant number of particles, pressure, and temperature) with Parrinello–Rahman pressure coupling at 1 bar with a compressibility of 4.5 × 10^−5^ bar^−1^ and a 2 ps time constant. Finally, the equilibrated system for T7RNAP model was subjected to 100 ns MD simulation with a time-step of 2 fs. All bonds were constrained using LINCS (Linear Constraint Solver) algorithm. The quality of the simulated systems at different pH strengths were analyzed by RMSD (root mean square deviation) and RMSF (root mean square fluctuation) plots. Various snapshots of T7RNAP were extracted from the trajectories (pH 5 and 7.9) at every 20 ns and checked for their consistency in protonation/deprotonation states with those assigned to the initial structure.

### Principal component and free energy landscape analysis

Principal Component Analysis (PCA) or Essential Dynamics^37, 38^ was carried out by generating a covariance-matrix of the atomic fluctuations. This covariance matrix was diagonalized to extract a set of eigenvectors and eigenvalues that reflect the combined motion of the molecule. Eigenvectors represent the direction of motion, whereas the corresponding eigen values represent the amplitudes in those directions. The GROMACS in-built utilities “g_covar” was used to generate the covariance matrix using protein backbone as reference structure for the rotational fit and “g_anaeig” was used to analyze and plot the eigenvectors. Majority of the motions (>90%) are represented by less than ten eigenvectors that illustrate the relevant combined motions within an atomic system. The first twenty projection eigenvectors of T7RNAP at different pH environments were retrieved and analyzed for their cosine distribution. The cosine values describe whether the MD simulation time interval used to extract the concerned trajectory is sufficient or not to represent the free energy landscape^39, 40^. After retrieving the suitable principal coordinates, the free energy landscapes (FELs) were generated for each simulated systems to identify and extract their near native conformations.

### Domain motion analysis

Conformational changes observed among the stable structures of T7RNAP at different pH values due to domain motions were analyzed by DynDom^25^. DynDom analyses conformational change in the protein for dynamic domains, hinge axes and hinge-bending regions. The procedure was performed in three consecutive steps. First, the dynamic domains were identified; second, the inter-domain screw axes were determined; and final step involved identification of the inter-domain bending regions. In order to visualize the direction of movements in each conformation at different pH conditions, the porcupine plots were generated for the MD trajectories using the ProDy interface of normal mode wizard (NMW) of VMD^41^.

### T-pad analysis

T-pad analysis^27^ tracks the protein angular dispersion of the angle ω (PADω) to quantitatively analyze local fluctuations and transitions of individual residues. These fluctuations and transitions in turn characterize their time-dependent properties from NMR structural ensembles or molecular dynamics trajectories. Although the circular spread of the Ramachandran angles Φ or ψ (CSΦ or CSψ) has been used earlier to analyze the plasticity of the protein backbone, circular spread does not completely account for the backbone conformation of the protein. PADω overcomes the limitations of Ramachandran angles and has been successfully used^42, 43^ to quantify the plasticity of protein backbone residues. PADω is a function of ω (=Φ + ψ), and hence dependent on both Ramachandran angles. Unlike torsion angle Cα−C−N−Cα in a peptide bond (CSω), PADω allows quantitative comparisons among residues and among proteins by keeping a narrow range of ω between 0° and 180°. T-pad analysis identifies fluctuations (F), long transitions (T) and short transitions (t). A fluctuation (F) is attributed to the fluctuations along a given direction and those along two separate directions are identified as a transition. The difference in long and short transition depends on PAIω (a function of CSω and the Angular Transition Index ATIω). The transitions having PAIω between 30° and 60° are attributed as long transitions and those between 60° and 90° are identified as short transitions. Detailed theory of T-pad tool has been reported by Caliandro *et al*.^27^.

### TEM and Single particle analysis of T7RNAP

T7RNAP was procured from New England Biolabs, Massachusetts, United States. The sample was diluted with its referred buffer (50 mM Tris-HCl, 100 mM NaCl, 20 mM β-ME and 1 mM EDTA) to a suitable concentration and pH was adjusted subsequently. Finally, T7RNAP in buffer at a suitable concentration was negatively stained on a freshly glow-discharged carbon-coated 400 mesh copper grid (Electron Microscopy Sciences, USA) with a 1% (w/v) aqueous solution of uranyl acetate.

Micrographs containing a good spread of the particles were collected using Carl Zeiss LIBRA 200 FE-HR transmission electron microscope (TEM) operated at 200 kV and at 50kX magnification. Images from the TEM were collected with the integrated slow scan charge coupled device (SSCCD) Camera with image size (2 K × 2 K).

Three-dimensional reconstruction of the single particles used the EMAN 1.9 software package^44^. The particles were collected from micrographs using “Boxer” program of EMAN 1.9. The particles were then used to generate class averages using EMAN’s “refine2d”routine. An initial three-dimensional model was calculated from a set of selected noise-free class averages. A convergent structure was obtained by refining the particle dataset over several iterations. A Fourier shell correlation (FSC) = 0.5 criterion was applied to determine the resolution. The convergence was examined by comparing the projections of the model with corresponding class averages. A molecular mass of 99 kDa was used for the surface-rendering threshold of the 3D structure. Three-dimensional reconstructions were visualized using the UCSF Chimera software package^45^ and fitting of simulated structures into the density map was performed by Mod-EM^46^, a density fitting module of MODELLER package^47^. The best fit between the probe model and the EM density map was obtained by changing the position of the model so as to maximize the cross-correlation between the probe density and the EM density. The cross-correlation was measured by a normalized fitting score.

### Docking with heparin and GTP

To check the different interaction affinities of the active site region of T7RNAP at different pH strengths, we docked heparin and GTP (Guanosine triphosphate) to the catalytic cavity.

Heparin, a known inhibitor at the active site of T7RNAP^48^, was collected from PubChem (PubChem CID: 444410). Heparin was energy minimized using LigPrep tool with an OPLS_2005 force field. Their ionization states were generated at pH 7.0 ± 2.0 using Ionizer in LigPrep. Specific chiralities were retained during ligand preparations and stereoisomers per ligand were retained at a minimum value of 1, as these compounds have too many atoms and are too flexible. The T7RNAP model was optimized by the protein preparation wizard and the grid box enclosed the active site residues (Palm sub-domain: Asp 537, His 811 and Asp 812 and Finger sub-domain: Lys 631, Tyr 639). Then, the prepared heparin was docked to the active site of T7RNAP using Glide XP module of Maestro 9.2 suite. While docking, flexible docking option was selected. Ring sampling energy window was kept at 2.5 kcal/mol. For energy minimization, distance-dependent dielectric constant was kept at its default value of 0.2 and maximum number of minimization steps (used by the conjugate gradient minimization algorithm) was kept at its default value of 100.

The structure of GTP was collected from PubChem (PubChem CID: 6830). The same protocol was applied for ligand preparation and docking as given above, except that during ligand preparation stereoisomer generation was kept at maximum value 32.

### Docking with T7 Promoter

To study the effect of variable pH on the interactions between T7RNAP and promoter DNA, we have used HADDOCK (High Ambiguity Driven protein-protein DOCKing) server^29^ for data-driven biomolecular docking. The docking was performed using the structures of T7RNAP obtained from the previous step at different pH strength and T7 promoter from the T7RNAP+T7 promoter complex (PDBID: 1CEZ)^30^. HADDOCK integrates restraints from mutagenesis and NMR chemical shift data in protein docked complexes which were then converted into a series of Ambiguous Interaction Restraints (AIR). The docked complexes are ranked on the basis of sum of electrostatics, van der Waals, and AIR energy terms. Docking in HADDOCK was performed in three major steps that involve rigid body docking through energy minimization, followed by refinement through simulated annealing of the best complexes, and finally refinement of the best docked complexes using the 8 Å explicit solvent layers (TIP3P). The positions for interacting residues for T7RNAP and promoter DNA were obtained from the crystal structure of T7RNAP-T7 promoter complex. The interaction pattern of T7RNAP+T7 promoter complexes were generated by NUCPLOT tool^49^.

## Electronic supplementary material

Supplementary Information
