# Supplementary material for: An in-silico glimpse into the pH dependent structural changes of T7 RNA polymerase: a protein with simplicity
Source: Sci Rep. 2017 Jul 24;7:6290. doi: 10.1038/s41598-017-06586-1 (PMC5524818; doi:10.1038/s41598-017-06586-1)
Supplement: Supplementary file 1 — Supplementary Information [file 41598_2017_6586_MOESM1_ESM.doc]

**Supplementary information**

**An *in-silico* glimpse into the pH dependent structural changes of T7 RNA polymerase: a protein with simplicity**

Subhomoi Borkotoky ^a^, Chetan Kumar Meena ^a^, Gopalkrishna M. Bhalerao ^b^, Ayaluru Murali ^a^*

^a^ Centre for Bioinformatics, School of Life Sciences, Pondicherry University, Puducherry-605014, India

^b^ UGC-DAE Consortium for Scientific Research Kalpakkam Node, Kokilamedu, Tamilnadu-603104, India

* Corresponding author: [murali@bicpu.edu.in](mailto:murali@bicpu.edu.in)


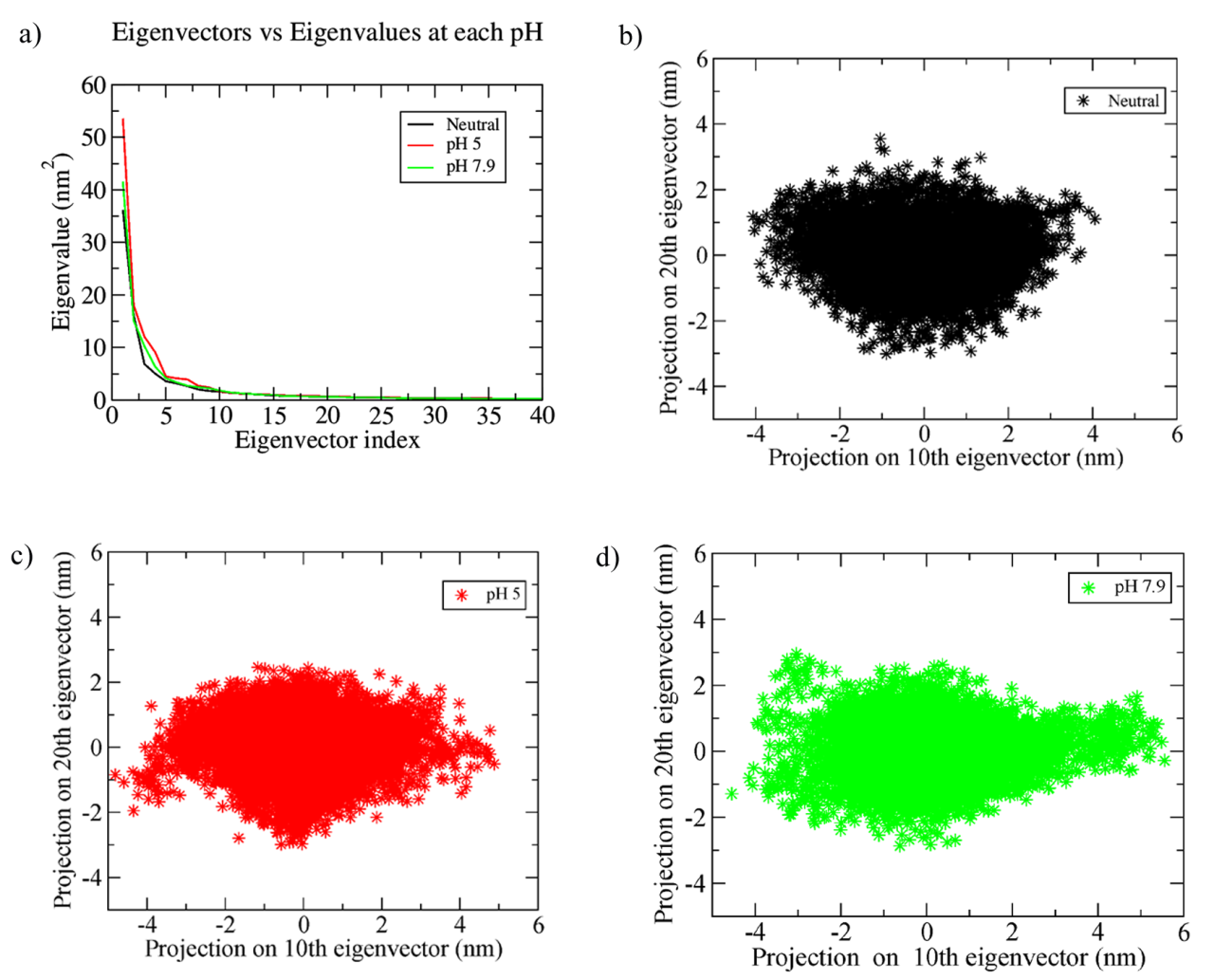


Supplementary Figure S1: a) Eigenvalues for T7RNAP at each pH shown in decreasing order of magnitude and obtained from the backbone coordinate covariance matrix as a function of the eigenvector index. The plots of the projections of the trajectory onto the planes defined by the tenth and twentieth eigenvectors from the backbone coordinate covariance matrix for T7RNAP at b) pH 7, c) pH 5 and d) pH 7.9.


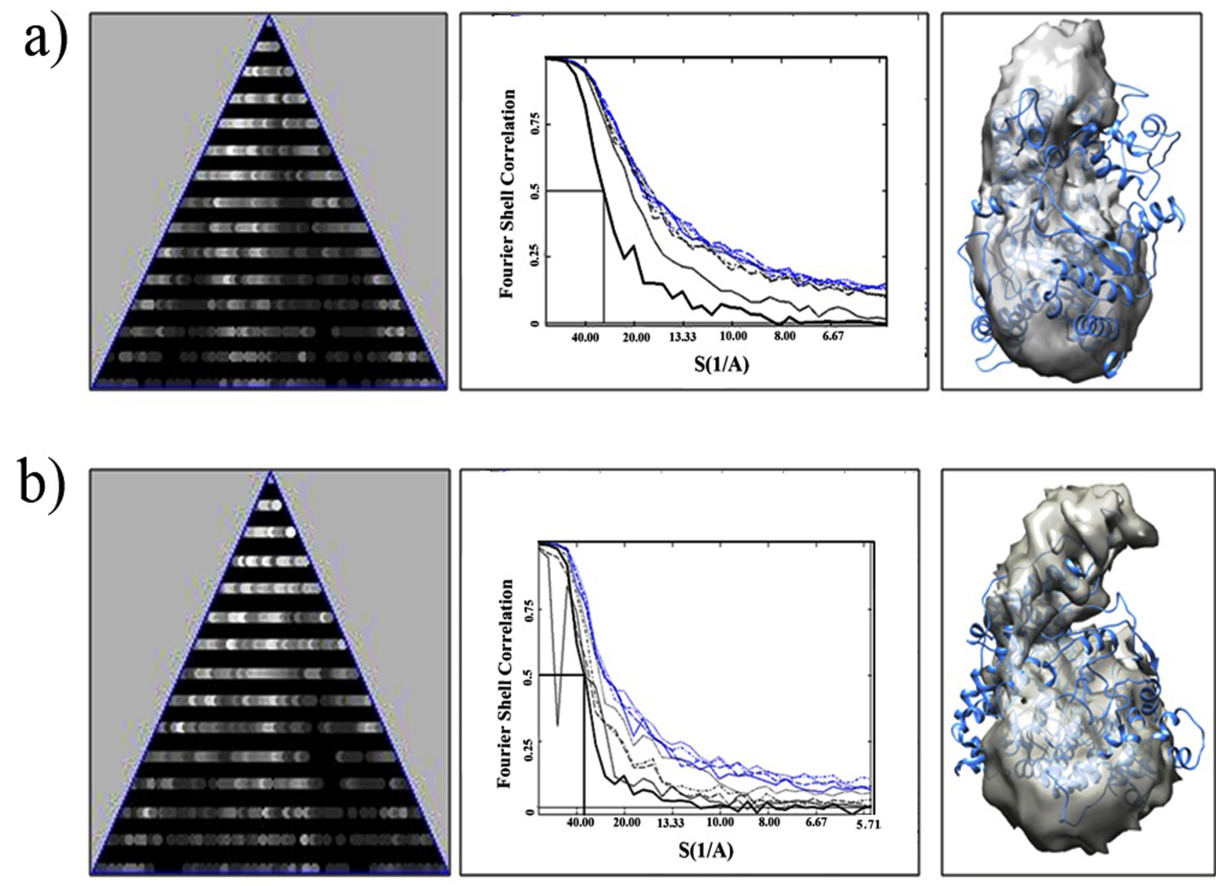


Supplementary Figure S2: Sections of TEM micrographs of negatively stained T7RNAP (inset shows the representative views of the average of several particles – not to scale), the triview map, FSC curves and fitting studies of simulated T7RNAP a) at pH 7.9 and b) at pH 5.


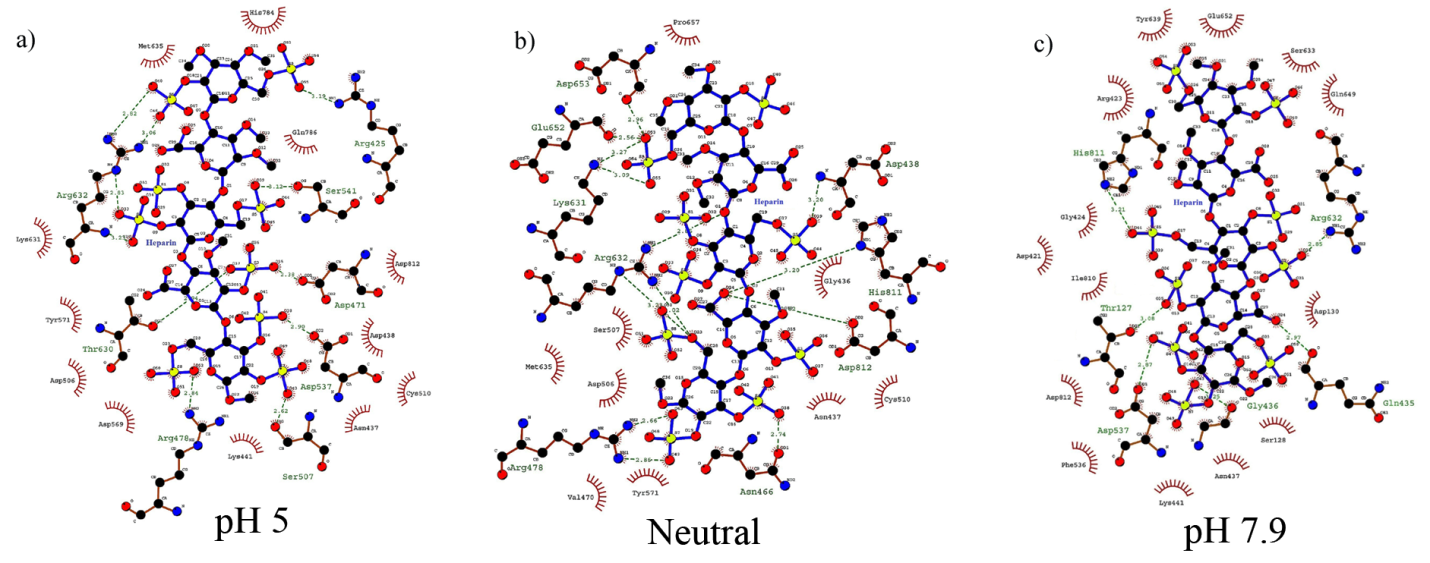


Supplementary Figure S3: Two dimensional representations of docked complexes of T7RNAP and Heparin at a) pH 5, b) Neutral and c) pH 7.9. The dotted lines denote H-bond, while the arcs with spokes denote hydrophobic interactions.


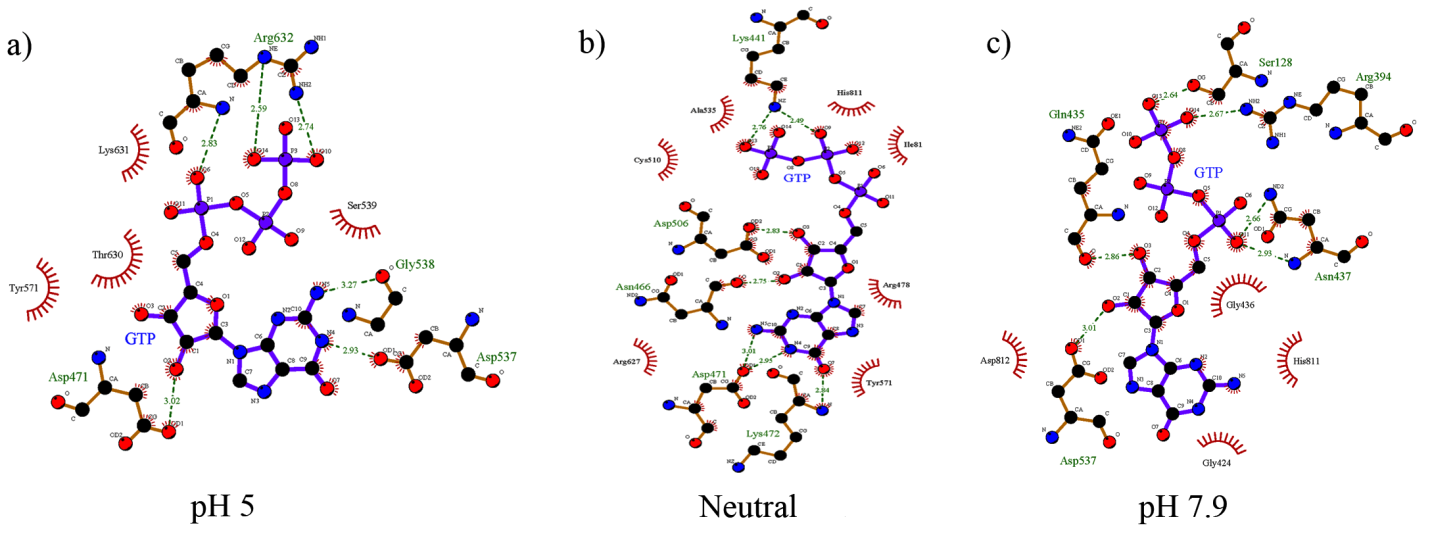


Supplementary Figure S4: Two dimensional representations of docked complexes of T7RNAP and GTP at a) pH 5, b) Neutral and c) pH 7.9. The dotted lines denote H-bond, while the arcs with spokes denote hydrophobic interactions.


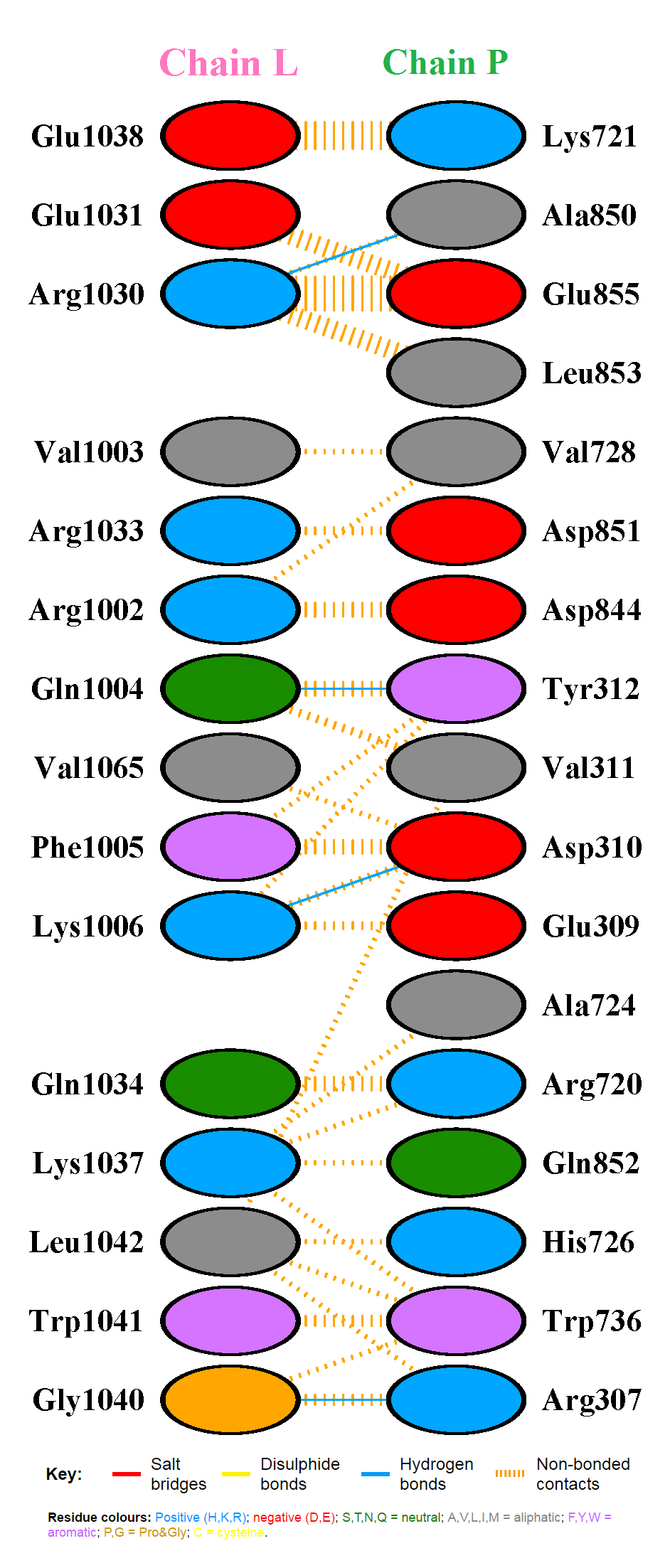


Supplementary Figure S5: Interaction pattern of T7RNAP with T7 lysozyme from the PDB entry 1ARO (T7RNAP-T7L complex). The pattern was generated by PDBsum server.


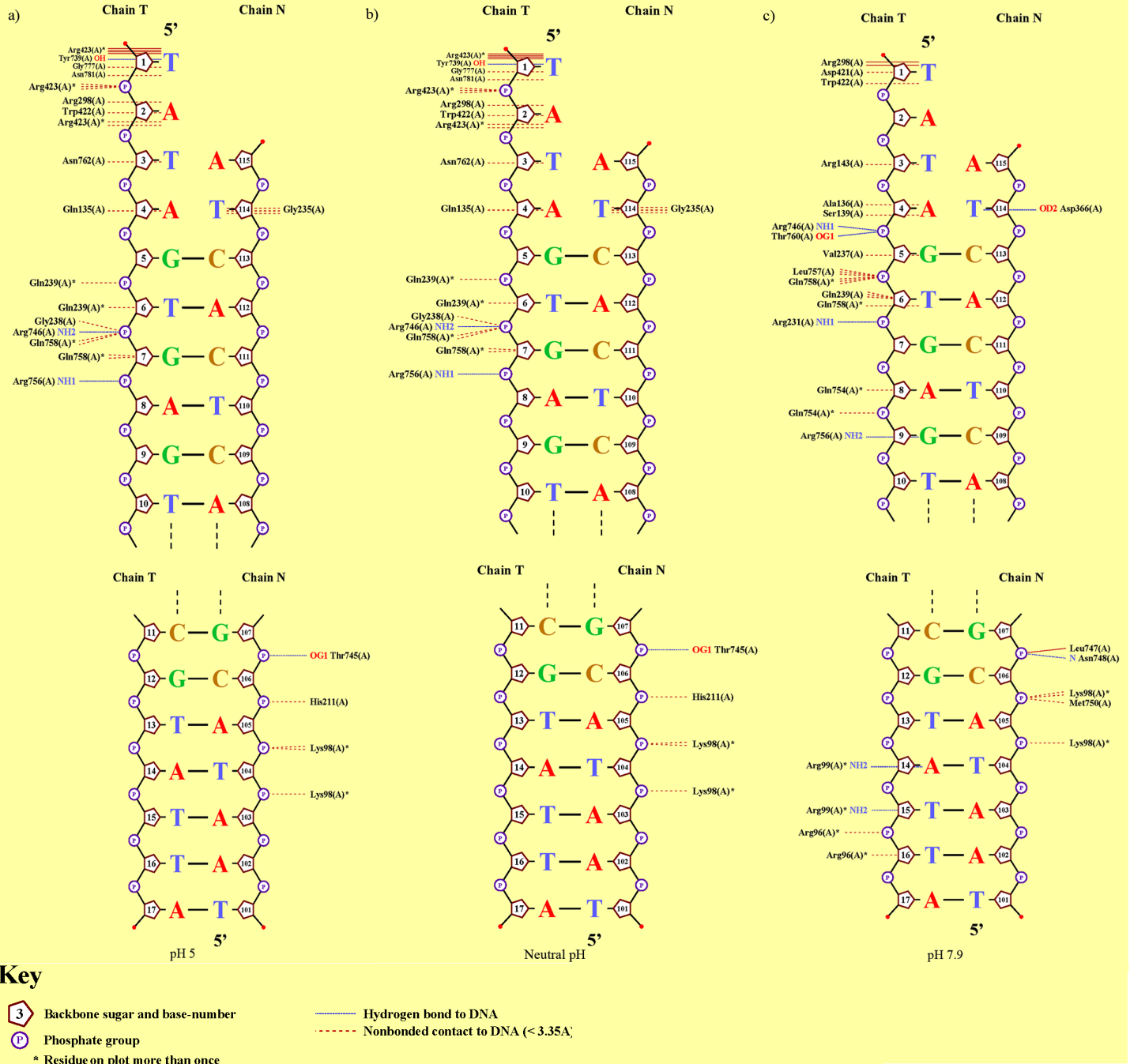


Supplementary Figure S6: Interaction plot of T7 promoter with T7RNAP at different pH viz. a) pH 5, b) Neutral and c) pH 7.9. Chain T represents template strand and chain N represents non-template strand.


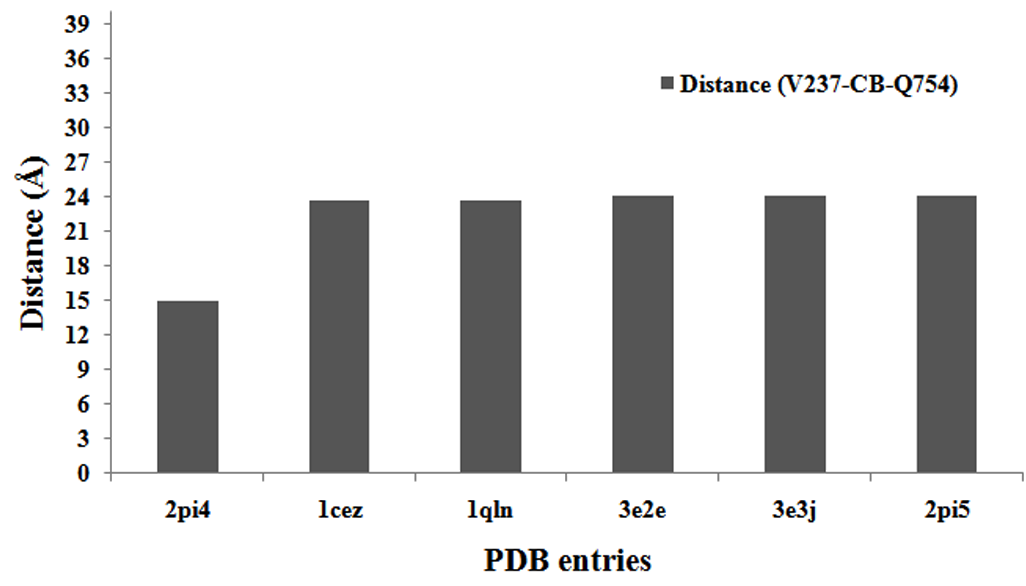


Supplementary Figure S7: Comparison of distance between Valine 237 and Glutamine 754 of two promoter interacting loops of N-terminal domain and finger sub-domain respectively among six PDB entries of T7RNAP (complexed with DNA).

Supplementary Table 1. T-pad angles of active site residues at each pH showing long transition (T), short transition (t) and fluctuation (F).

| **Residues** | **pH 5** | | | **pH 7.9** | | | **Neutral** | | |
| --- | --- | --- | --- | --- | --- | --- | --- | --- | --- |
|  | **T** | **t** | **F** | **T** | **t** | **F** | **T** | **t** | **F** |
| **536** | - | - | 32.64º | 42.27º | - | - | 45.16º | - | - |
| **537** | - | - | 41.31º | - | - | 45.38º | - | - | 50.25º |
| **538** | - | - | 32.34 º | - | - | 50.01º | - | 108.56º | - |
| **539** | - | 72.03º | - | 79.5 º | - | - | - | 132.74º | - |
| **540** | - | 59.32º | - | 61.61º | - | - | 47.91º | - | - |
| **626** | - |  | 138.42 º | - | 58.36º | - | 100.93º | - | - |
| **627** | - | 121.03º | - | - | 58.06º | - |  | 53.8º | - |
| **628** | - | 102.95º | - | 66.54º |  | - |  | 38.27º | - |
| **629** | 91.0 º | - | - | - | 68.14º | - | 51.28º | - | - |
| **630** | 98.32º | - | - | - | - | 53.18º | 47.89º | - | - |
| **631** | - | 98.19º | - | - | - | 37.26º |  | 64.21º | - |
| **632** | - | 96.2º | - | 42.25º | - |  | 57.4º |  | - |
| **633** | 54.28º |  | - | - | - | 27.24º | - | 29.29º | - |
| **634** | - | - | 24.91º | - | - | 34.7 º | - | - | 24.73º |
| **635** | - | - | 26.24º | 44.67º | - | - | - | - | 24.18º |
| **636** | - | - | 33.3 º |  | 69.21º | - | - | - | 22.31º |
| **637** | - | - | 24.74º | 82.14º |  | - | - | - | 27.69º |
| **638** | - | - | 30.6º | - | 61.65º | - | - | - | 37.44º |
| **639** | - | - | 40.94º | - | 53.83º | - | - | - | 40.01º |
| **810** | - | - | 18.88º | - | - | 19.05º | - | - | 18.51º |
| **811** | 31.98º | - | - | - | - | 34.42º | - | - | 34.27º |
| **812** | 35.6º | - | - | 45.64º | - | - | - | - | 31.97º |
| **813** |  | - | 34.98 | 47.95 | - | - | - | - | 28.56 |

Supplementary Table 2. T-pad angles of T7RNAP sub-domains at pH 5 showing long transition (T), short transition (t) and fluctuation (F).

| **Residues** | **Finger** | | | **Residues** | **N-terminal** | | | **Residues** | **Thumb** | | |
| --- | --- | --- | --- | --- | --- | --- | --- | --- | --- | --- | --- |
|  | **T** | **t** | **F** |  | **T** | **t** | **F** |  | **T** | **t** | **F** |
| **607** | 64.43º | - | - | **52** | - | - | 18.69º | **363** | - | - | 34.8º |
| **608** | - | 114.13º | - | **53** | - | - | 21.27º | **364** | 61.93º | - | - |
| **609** | 68.54º | - | - | **54** | - | - | 23.19º | **365** | 97.9º | - | - |
| **610** | - | 58.91º | - | **55** | - | - | 28.42º | **366** | - | - | 58.78º |
| **611** | - | 76.26º | - | **56** | - | 81.51º | - | **367** | - | - | 42.62º |
| **612** | - | 83.49º | - | **57** | - | 90.66º | - | **368** | 56.11º | - | - |
| **613** | - | 62.77º | - | **58** | 50.24º | - | - | **369** | - | 83.3º | - |
| **614** | 88.51º | - | - | **59** | 94.75º | - | - | **370** | 70.76º | - | - |
| **615** | 122.48º | - | - | **60** | 128.1º | - | - | **371** | - | - | 24.98º |
| **616** | - | 50.54º | - | **61** | - | 111.3º | - |  | | | |
| **617** | - | - | 28.12º | **62** | - | - | 159.2º |  |  |  |  |
| **618** | - | - | 29.72º | **63** | 84.72º | - | - |  |  |  |  |
| **619** | - | - | 26.17º | **64** | 86.59º | - | - |  |  |  |  |
| **620** | 30.17º | - | - | **65** | - | 63.04º | - |  |  |  |  |
| **711** | 44.82º | - | - | **95** | - | - | 46.15º |  |  |  |  |
| **712** | 54.99º | - | - | **96** | - | 107.5º | - |  |  |  |  |
| **713** | - | - | 57.85º | **97** | - | 146.1º | - |  |  |  |  |
| **714** | - | 70.37º | - | **98** | - | - | 47.41º |  |  |  |  |
| **715** | - | 98.2º | - |  | | | |  |  |  |  |
| **716** | - | 111.27º | - |  |  |  |  |  |  |  |  |
| **717** | - | - | 75.38º |  |  |  |  |  |  |  |  |
| **718** | - | - | 128.4º |  |  |  |  |  |  |  |  |
